# Supplementary material for: Anti-TCP1 Antibody Is a Potential Biomarker for Diagnosing Systemic Lupus Erythematosus
Source: Int J Mol Sci. 2024 Aug 7;25(16):8612. doi: 10.3390/ijms25168612 (PMC11354590; doi:10.3390/ijms25168612)
Supplement: Supplementary file 1 [file ijms-25-08612-s001.zip › ijms-3123530-supplementary.pdf]

Supplementary Table S1. Clinical Characteristics of Patients with SLE, RA, BD, SSc, and NCs

	SLE (n=251)	RA (n=25)	BD (n=28)	SSc (n=30)	NCs (n=50)
Age (SD), years	42.4 (11.7)	46.1 (11.5)	45.2 (9.7)	49.5 (11.7)	40.4 (8.4)
Female, no. (%)	239 (95.2)	25 (100)	21 (75)	25 (83.3)	44 (88)
Disease duration (SD), years	12.9 (6.9)	10.4 (5.7)	15.4 (6.5)	11.2 (5.7)	
ANA positive, no. (%)	213 (84.9)				
Anti-dsDNA Ab positive no. (%)	94 (37.5)				
C3, mg/dL (SD)	91.6 (67.6)				
C4, mg/dL (SD)	18.8 (12.5)				

SLE: systemic lupus erythematosus, RA: rheumatoid arthritis, BD: Behçet's disease, SSc; systemic sclerosis, NCs: normal controls, SD: standard deviation; ANA: antinuclear antibody; Anti-ds DNA Ab: anti-double stranded DNA; C3: complement 3; C4: complement 4
